# *Mycobacterium novocastrense*–associated Pulmonary and Wound Infections

**DOI:** 10.3201/eid1703.101400

**Published:** 2011-03

**Authors:** Hasan Shojaei, Abodolrazagh Hashemi, Parvin Heidarieh, Abass Daei Naser

**Affiliations:** Author affiliations: Isfahan University of Medical Sciences, Isfahan, Iran (H. Shojaei, A. Hashemi, A.D. Naser);; Tehran University of Medical Sciences, Tehran, Iran (P. Heidarieh)

**Keywords:** Mycobacteria, Mycobacterium novocastrense, nontuberculous mycobacteria, NTM, 16S rDNA, *hsp*65, tuberculosis and other mycobacterial diseases, bacteria, letter

**To the Editor:** Although the clinical role of nontuberculous mycobacteria has long been appreciated (*1*), the high endemicity of tuberculosis (TB) in developing countries has overshadowed the emergence of these organisms. They are simply dismissed as being contaminants or are misidentified as *Mycobacterium tuberculosis* (*2*).

No report on *M. novocastrense* has been published since its original description in 1997 (*3*), except for a study in France by N’Guessan et al. (*4*). That study initiated speculation about the possible role of this bacterium in the etiology of human infection. We report isolation of *M. novocastrense* in 2 independent clinical cases—1 from tissue biopsy specimen of an apparently healthy adult, the other from bronchoalveolar lavage of an HIV-infected patient—that will cast light on the clinical relevance of this rare species.

Case-patient 1, a 60-year-old woman, was referred to a hospital because of high fever, productive cough, thoracic pain, and noticeable weight loss. After her husband’s AIDS-related death in a state prison, HIV infection had been diagnosed in this case-patient. At admission, laboratory testing showed negative tuberculin skin test result, lymphopenia, an elevated C-reactive protein level of 76 mg/L, an erythrocyte sedimentation rate of 73 mm/h, a viral load of 500 copies/mL, and negative blood culture for bacterial growth. Her outpatient records indicated that she empirically was given numerous courses of antimicrobial drugs because of a provisional diagnosis of bacterial pneumonia, but her condition did not improve. Subsequent hospital referral was prompted by worsening of her symptoms. With a diagnosis of suspected TB, bronchoalveolar lavage fluid was collected. Direct microscopic examination of the specimen showed acid-fast bacilli with subsequent formation of typical colonies of a relatively rapidly growing photochromogenic *Mycobacterium* spp. on Löwenstein-Jensen (LJ) medium. The patient was treated with amikacin, and her condition markedly improved.

Case-patient 2, a 23-year-old woman, sought care for a 6-month history of soft tissue swelling in her left leg resulting from an accidental injury in a paddy field. The lesion had not been treated and subsequently increased in size. The large nodule self-ruptured and excreted yellow-pale pus. She was prescribed minocycline by her general practitioner, but the lesion did not resolve. When the patient arrived at the hospital, her clinical and laboratory assessments were normal. She had negative test results for HIV, hepatitis C, and hepatitis B infections. Erythrocyte sedimentation rate was 255 mm/h. Culture of a biopsy specimen from the cutaneous lesion on ordinary culture media was negative, although microscopic observation of the drainage fluid showed acid-fast bacilli. This finding was confirmed by a positive culture on LJ medium. A repeat specimen resulted in isolation of the same organism in pure culture. The patient was given amikacin and fully recovered in <1 month.

The isolates, i.e., HNTM1 and HNTM10, were subjected to preliminary identification and susceptibility to common antimycobacterial agents for rapidly growing mycobacteria according to standard procedures (*5**,**6*). The isolates were then subjected to molecular identification, which included PCR amplification of a genus-specific region of the 65-kDa heat shock protein (*hsp*) gene (*7*) and the direct sequence analysis of 16S rDNA, *hsp*65, and *rpo*B genes as described (*3**,**8**,**9*). GenBank accession numbers for the gene sequences of HNTM1 as a representative isolate determined in this study are HM807280–HM807282.

The isolates we observed by acid-fast staining of the specimens and recovered on LJ medium were a yellow pigmented photochromogenic species that grew rapidly at 25°C, 37°C, and 42°C. They grew on MacConkey agar without crystal violet and LJ medium containing 5% NaCl; were positive for semiquantitative catalase, arylsulfatase activity in 14 days, and nitrate reduction; and were negative for urease activity, niacin production, tellurite and Tween hydrolysis, heat-stable (68°C) catalase, and iron uptake. They were susceptible to amikacin, clarithromycin, doxycycline, sulfamethoxazole, streptomycin, imipenem, ciprofloxacin, isoniazid, and ethambutol but resistant to rifampin.

The PCR amplification of a 228-bp genus-specific fragment of the *hsp*65 gene reliably confirmed that the isolates belonged to the genus *Mycobacterium*. The almost complete 16S rDNA gene sequence (1,476 bp) and partial sequences of *hsp65* and *rpoB* genes of the isolates showed the highest similarity of 99.86%, 99.45%, and 99.7% with that of *M. novocastrense* reference strain. These values correspond to 2-nt differences for each gene.

Our report of 2 independent clinical cases might support evidence the clinical relevance of *M. novocastrense*. Our findings show that *M. novocastrense*, however rare its incidence might be, can cause infection in healthy and immunocompromised patients. However, because of the complexity of identifying nontuberculous mycobacteria, emphasis should be placed on the quality of regional laboratories for TB in developing countries to differentiate isolates to the species level.

## References

[1] Falkinham JO III. Epidemiology of infection by nontuberculous mycobacteria. Clin Microbiol Rev. 1996;9:177–215. 8964035 10.1128/cmr.9.2.177 PMC172890

[2] Jarzembowski JA, Young MB. Nontuberculous mycobacterial infections. Arch Pathol Lab Med. 2008;132:1333–41. 18684037 10.5858/2008-132-1333-NMI

[3] Shojaei H, Magee JG, Freeman R, Yates M, Horadagoda NU, Goodfellow M. *Mycobacterium novocastrense* sp. nov., a rapidly growing photochromogenic *Mycobacterium.* Int J Syst Bacteriol. 1997;47:1205–7. 10.1099/00207713-47-4-1205 9336929

[4] N’Guessan K, Vincent V, Nahoua G, Kamate M, Dosso M. *Mycobacterium novocastrense* isolated from sputum: contamination or pulmonary infection? [in French]. Med Mal Infect. 2006;36:180. 16488568 10.1016/j.medmal.2005.11.011

[5] Kent PT, Kubica GP. Public health mycobacteriology: a guide for the level III laboratory. Atlanta: Centers for Disease Control, US Department of Health and Human Services; 1985.

[6] National Committee for Clinical Laboratory Standards. Susceptibility testing of mycobacteria, nocardiae, and other aerobic actinomycetes: approved standard. Wayne (PA): The Committee; 2003. 31339680

[7] Khan IU, Yadav JS. Development of a single-tube, cell lysis-based, genus-specific PCR method for rapid identification of mycobacteria: optimization of cell lysis, PCR primers and conditions, and restriction pattern analysis. J Clin Microbiol. 2004;42:453–7. 10.1128/JCM.42.1.453-457.2004 14715804 PMC321669

[8] Kim H, Kim SH, Shim TS, Kim MN, Bai GH, Park YG, Differentiation of *Mycobacterium* species by analysis of the heat-shock protein 65 gene (*hsp*65). Int J Syst Evol Microbiol. 2005;55:1649–56. 10.1099/ijs.0.63553-0 16014496

[9] Adékambi T, Colson P, Drancourt M. *rpo*B-based identification of nonpigmented and late-pigmenting rapidly growing mycobacteria. J Clin Microbiol. 2003;41:5699–708. 10.1128/JCM.41.12.5699-5708.2003 14662964 PMC308974

